# Oil palm concessions in southern Myanmar consist mostly of unconverted forest

**DOI:** 10.1038/s41598-019-48443-3

**Published:** 2019-08-15

**Authors:** Keiko Nomura, Edward T. A. Mitchard, Genevieve Patenaude, Joan Bastide, Patrick Oswald, Thazin Nwe

**Affiliations:** 1 University of Edinburgh, School of GeoSciences, Edinburgh, EH9 3FF United Kingdom; 2 Centre for Development and Environment (CDE), University of Bern, Hallerstrasse 10, 3012 Bern, Switzerland; 3 OneMap Myanmar, No. E2 New University Yeik Mon, New University Avenue, Bahan Township, Yangon, Myanmar; 4 Biodiversity Conservation Group, Center for Integrative Conservation, Xishuangbanna Tropical Botanical Garden, Chinese Academy of Sciences, Menglun, Xishuangbanna, Yunnan 666303 China

**Keywords:** Sustainability, Climate-change mitigation

## Abstract

The increased demand for palm oil has led to an expansion of oil palm concessions in the tropics, and the clearing of abundant forest as a result. However, concessions are typically incompletely planted to varying degrees, leaving much land unused. The remaining forests within such concessions are at high risk of deforestation, as there are normally no legal hurdles to their clearance, therefore making them excellent targets for conservation. We investigated the location of oil palm plantations and the other major crop – rubber plantations in southern Myanmar, and compared them to concession boundaries. Our results show that rubber plantations cover much larger areas than oil palm in the region, indicating that rubber is the region’s preferred crop. Furthermore, only 15% of the total concession area is currently planted with oil palm (49,000 ha), while 25,000 ha is planted outside concession boundaries. While this may in part be due to uncertain and/or changing boundaries, this leaves most of the concession area available for other land uses, including forest conservation and communities’ livelihood needs. Reconsidering the remaining concession areas can also significantly reduce future emission risks from the region.

## Introduction

Oil palm (*Elaeis guineensis Jacq*.) plantations have increased in area from 3.2 million ha in 1970 to 21 million ha in 2017^1^. Most of the land for these plantations has come from the clearance of tropical forest, thereby contributing large CO_2_ emissions into the atmosphere, and thus intensifying climate change^2–6^. The clearance of forest, along with this climate change, will together have further detrimental impacts on biodiversity and cause a reduction in the provision of ecosystem services^5–7^. Oil palm has also transformed livelihoods across the tropics, especially in Southeast Asia, where it has become the main export crop from countries such as Indonesia and Malaysia^1,8^. One can easily comprehend why: oil palm is exceptionally productive in optimal conditions, producing five times more oil per hectare than any other oil crop^9,10^. The resulting low price has created an increasing global demand, and the use of palm oil has expanded beyond food and personal care products to biofuel^11,12^. However, much of the area where oil palm now grows was until recently forest: an estimated 45% of oil palm in Southeast Asia grows on land that was forest in 1989^5^. In Kalimantan, Indonesia, 47% of lands converted to oil palm from 1990 to 2010 were previously intact forests^13^. There exists some pressure to increase the sustainability of palm oil by reducing deforestation led to the establishment of certification schemes. However, the continued growth of palm oil production is also driven by demand from developing economies, where price sensitivity trumps sustainability^14^.

Although the relationship between the expansion of oil palm plantations and declining forest area is well established^2–6,15^, the proportion of unplanted areas within oil palm concessions is not well known. According to the report “Hidden Lands, Hidden Risks?” by the Zoologist Society of London^16^, out of 8.6 million ha of land assigned for oil palm plantations (reported by the 50 largest oil palm companies), 1.4 million ha were of unclear use or remained unplanted. Meanwhile, 35 companies did not report unplanted areas. Other regional figures suggest this may be the tip of the iceberg: one study found that there may be as much as 1.7 million ha of standing forests in oil palm concessions in Indonesia alone^4^. If cleared, this would amount to emissions between 356–639 Tg C^4^. Another study found that that approximately 79% of oil palm concessions in Kalimantan, Indonesia have still not been planted^13^. If planted, 9 million ha of tropical forests (41% of intact forests) will be converted, resulting in 3.6–4.5 Pg C^13^.

The potential for conservation to change the fate of these as-yet-unconverted forests is significant. While there are typically lags between the time when the concession is granted and clearing the land and planting the crop is started, this cannot account for the degree of unplanting observed. Clearly, the development of oil palm is influenced by market conditions and the political environment, both at a national and local scale^3,17^. Further, some concession areas present high social and/or environmental risks and therefore remain unexploited^16^. For example, in Myanmar, the location of this study, there are conflicts over land ownership and access in some concession areas that are controlled by an insurgent group or occupied by wildlife (e.g. elephants)^18–20^. The conflict also involves communities that have returned to the region after the ceasefire agreement was signed in 2012, where the area has since been allocated for oil palm^19,21^. In addition, many concessions include lands that are simply unsuitable for plantations (e.g. steep slopes, lack of infrastructure for access)^18^. Furthermore, many of the companies who hold the concessions lack the resources, knowledge or even interests in investing in oil palm plantations^18,19^. While they engaged in logging where accessible, planting did not follow and large areas of concessions remain unused^18,19^. For the last few years, the government has been conducting oil palm land use assessment with the aim of allocating unused land to communities^22,23^.

**Figure 1:**
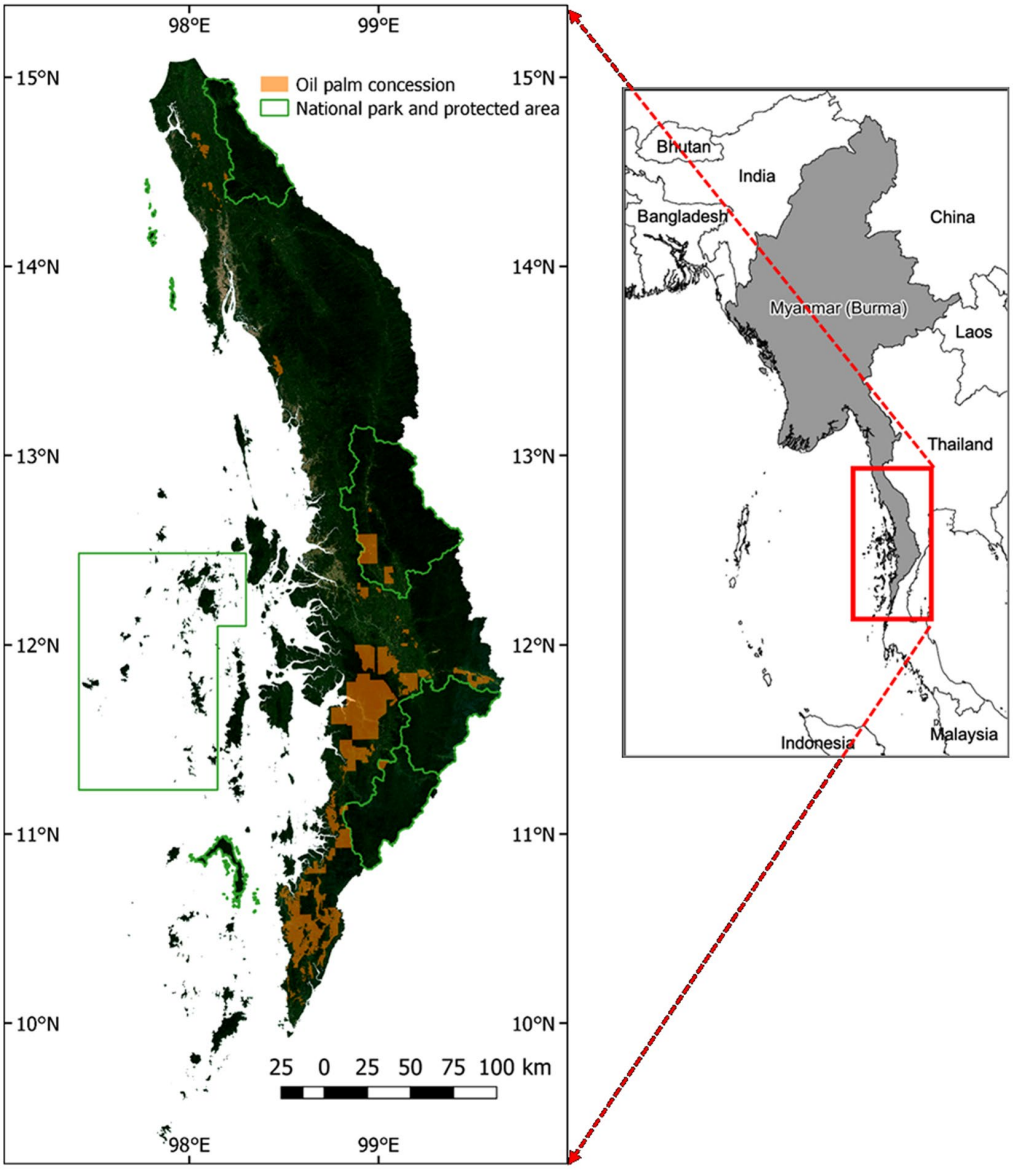
Tanintharyi region in Myanmar, Southeast Asia (right); oil palm concessions, national parks (designated and proposed), and protected areas in the region (left) as of 2018. Note that approximately 25,000 ha of concession area overlap with two proposed national parks (the Tanintharyi National Park and the Lenya National Park). The underlying map on the left is the Sentinel-2 cloud-free composite between November 2018 and January 2019.

Myanmar presents significant opportunities for conservation and sustainable development within oil palm concession areas. Although Indonesia is the largest oil palm producer today, its climate suitability for palm oil is projected to decrease significantly by 2050, while the climate suitability is projected to increase in Myanmar^24^. There are approximately 2 million ha of intact forests (estimates ranging from 1.9 million ha in 2016 and 2.3 million ha in 2014) in Southern Myanmar where oil palm concessions have been granted^25,26^. One reason for this is that prior to 1999, Myanmar had little history of oil palm industry development. Oil palm was introduced to the country in the 1920s^27^. Thereafter and from the 1970s on, oil palm plantations were developed in Tanintharyi, Mon, Kachin, and Rakhine states^27^. Yet, large scale development of oil palm plantations was only initiated in 1999, when it became a focus of the Myanmar government, with efforts concentrated in the southernmost part of the country: the Tanintharyi region, where the conditions were considered particularly favourable (Fig. 1)^18,28^. To meet domestic demand, and with the aim of going into an export industry, the government set a target of planting 202,343 ha by 2030 (500,000 ac, later increased to 700,000 ac (283,280 ha)), including about 63,000 ha of reserved forests)^18,20^. Under the then military regime, selected companies were tasked to operationalise the cultivation of oil palm. Since then, 401,814 ha of oil palm concessions have been allocated to 44 companies (Fig. 1), including some concessions overlapping with proposed national parks^18^. Of these, approximately 35% of the total concession areas (140,247 ha) are reported to have been planted (as of 2015)^18^. In Tanintharyi, the deforestation between 2001 and 2010 amounted to an estimated 164,200 ha^29^. The decline of forest extent was particularly high in one of the proposed national parks (Lenya) (98.0% to 95.2% between 2002 and 2016)^30^.

It is worth noting that oil palm is not the only major commodity crop in the region^31^. Tanintharyi also has large areas of rubber plantations, with their total extent the second largest of any region of the country^32^. Although concession data are not available as rubber plantations include smallholders, planted areas increased from 82,047 ha in 2008–2009 to 138,828 ha in 2015–2016, likely due to market liberalisations and an increase in the rubber price over the last two decades^19,32,33^.

Our study therefore investigates the current extent of oil palm and rubber plantations in Tanintharyi, Myanmar, and compares them to concessions and other boundaries. We estimated the area of oil palm as well as other land cover classes by conducting a machine learning classification on Sentinel-1 and Sentinel-2 satellite data from 2018–2019 using thousands of reference data points. Sentinel data are provided at a high resolution (~10 m for Sentinel-1 and 10–60 m for Sentinel-2) with very narrow bands that enable some differentiation of spectral responses from vegetation that was previously only possible using hyperspectral sensors (Sentinel-2’s B5-7, 8A), which is necessary to classify the region with complex landscapes with small patches of plantations and forests^34^. Furthermore, their frequent revisits (6/12-day for Sentinel-1, 5-day for Sentinel-2) made it possible to create a high-quality composite based on the average of many scenes, reducing radar speckle (S1), sun-angle, and seasonal effects (S2). The classification consists of six classes: mature oil palm, mature rubber trees, other trees, shrub, bare land, and water. By understanding the scale of oil palm plantations in Myanmar and identifying the total unplanted areas within concessions, our study provides critical information on the suitability and availability of land without the two crops that could be reassigned for other land use, including for communities to meet their livelihood needs and conservation of remaining forests. The impacts also transcend those related to forest cover and carbon: the area includes wildlife sanctuaries, national parks and other protected areas and is listed as a biodiversity hotspot. It hosts Sundaic flora, fauna as well as endemic and endangered species such as the Gurney’s Pitta (*Pitta gurneyi*), the Sunda pangolin (*Manis javanica*), and two recently discovered species of bent-toed geckos (*genus Cyrtodactylus*)^20,30,35^.

## Methods

### Data

Optical, radar, and elevation data were used to classify the Tanintharyi region into six classes: oil palm, rubber, shrub, other trees, bare land, and water, using Google Earth Engine. Sentinel-2 Level-1C (S2) data (optical) were obtained as image collections for the period between 01/11/2018 and 31/01/2019 (Table 1).

**Table 1:** Sentinel-2 and Sentinel-1 data used for classification.

Data	Period	Bands	Number of images
Sentinel-2 MSI: MultiSpectral Instrument, Level-1C	November 2018 to January 2019	B1–B12 [B1 and B10 were used for cloud detection only]	482
Sentinel-1 SAR GRD: C-band Synthetic Aperture Radar Ground Range Detected, log scaling	January 2018 to January 2019*	VV, VH	404

*January 2018 to March 2018 are from the ascending angle only; the remainder of the period are from both descending and ascending angles.

Cloudy pixels were processed using the cloud mask (QA60 band) provided in S2 data as well as a set of algorithms built to detect clouds using relevant B bands (B1, B2, B8, B10 and B11) (See Supplementary Information). These algorithms were customised for the region to create cloud-free image composites. The final image was produced by computing the mean of all bands in the 40 to 60 percentile range, and 10 bands (B2-B8A, B11-B12, all 10 or 20 m resolution) were selected for classification. In addition, two indices were included to detect vegetation through the greenness and texture: the normalised difference vegetation index (NDVI; Equation below); and the standard deviation of NDVI (moving window 5 × 5 pixels), both calculated at a 10 m resolution^36^. NDVI was selected over other indices (e.g. EVI, LSWI, SATVI) based on our previous study, which successfully classified similar landscapes in this region with high accuracy^34^. Including other indices did not result in significant changes in accuracy for our study. Furthermore, we avoided using closely correlated bands together in classifications as they can increase the chances of overfitting, and increase computation time, without increasing accuracy^37,38^.

$$NDVI=\frac{(B8-B4)}{(B8+B4)}$$

Sentinel-1 data (dual-polarization C-band Synthetic Aperture Radar) was obtained for 13 months: from January 2018 to January 2019 (only January 2018 to March 2019 for Ascending mode due to an artefact) (Table 1). The images for the region include both from descending and ascending angles at a 10 m resolution, and the mean and standard deviation of VH (vertical transmission; horizontal reception) and VV (vertical transmission; vertical reception) bands were used for classification. Slope was calculated using the elevation data from the Shuttle Radar Topography Mission (SRTM) at a 30 m resolution^39^. After adding the spectral and radar bands, NDVI, the texture index, and slopes, the images were scaled to a 20 m spatial resolution. The 20 m resolution was selected because of the ‘red edge’ bands (B5-7, B8A) in Sentinel-2 that cover narrow portions of the spectrum (<20 nm wide), which are useful in classifying visually similar tree crops^34^.

The reference data required for training and testing in classification were obtained from our previous study^34^ and by manual selection using high resolution (<2 m) data viewed in Google Earth Pro. Most of the reference data for oil palm, rubber, and shrub were from high resolution images in Google Earth taken in 2018, with some exceptions going back to 2016, where those areas were checked for any changes with annual tree loss data from the Global Forest Change^40^. The reference data for bare land and water classes were taken from the Sentinel-2 cloud-free composite (November 2018 to January 2019) as well as from high resolution images in Google Earth from 2017 and 2018. The remaining class, “other” covers the largest areas in the region as it includes intact forests, mixed forests, betel & cashew nut plantations, and any other vegetation on the ground. Due to the lack of recent high resolution images in some areas, a few samples for the other class were taken from the images in 2015, which were checked with the Global Forest Change as well as the database from the Intact Forest Landscapes^40,41^. The data were delineated as polygons and a stratified sampling method was used by randomly selecting 50% of pixels (20 × 20 m) in polygons for training and testing. A total of 170,916 pixels were collected as reference data, including 32,945 pixels for oil palm and 13,384 pixels for rubber (See Figs S1 and Table S1).

The oil palm concession area data were provided by OneMap Myanmar in October 2018 and used in the aggregated form^42^. Due to frequent changes, the data may include concessions that are cancelled or with unclear status. GIS data are digitised based on map information available in the land use permits: sketch maps, or maps drawn on old topographic one inch maps. OneMap Myanmar states that the data are provided as is, with all efforts made to produce a good dataset, but that the accuracy of the concession data is not guaranteed.

### Classification

A machine learning algorithm, Random Forest was used to perform the classification^43,44^. Two parameters for the Random Forest classifier, the number of classification trees and the number of prediction variables per node were set at 100 and 4, respectively. Accuracy did not increase beyond 100 trees, so this was used, and the number of prediction variables were set at the square root of input variables (~the square root of 17)^45^. After the classification, the pixels were filtered to represent the majority of the connected pixels at a 3 × 3 pixel neighbourhood window.

### Accuracy

50% of reference data were randomly selected per class and set aside for testing (85,381 pixels). The overall accuracy, user’s accuracy (UA), and producer’s accuracy (PA) were calculated for two areas. The overall accuracy rate indicates the proportion of the area mapped correctly. UA and PA are the proportion of the area mapped as a particular class. UA is about the probability of a pixel in the output map being that class in reality, while PA is about the probability of a pixel in the test dataset of a particular class being correctly mapped^46^.

### Area estimation

The areas for each class were estimated by adjusting for classification errors and biases by using the reference data and a standard method^46^. The classified pixels, after filtering, were counted for each class in the total area, and the proportion of the area mapped as the class was used to estimate the area for that class by multiplying it by the total area. Therefore, the final area estimates were based on the reference classification of each class and provided with 95% confidence intervals (See Tables S3 and S4).

## Results

We obtained an overall accuracy against independent test data of 94% when using satellite data to classify the region’s land cover into six classes (See the Methods section). The accuracy rates for oil palm and rubber ranged between 84–96% and 81–95%, respectively (See Table S2 for full error matrices, including user’s and producer’s accuracy). The uncertainty and bias inherent in the classification, estimated using independent data, was used to estimate bias-corrected area and 95% confidence intervals for each class (See Tables S3 and S4)^46^.

**Figure 2:**
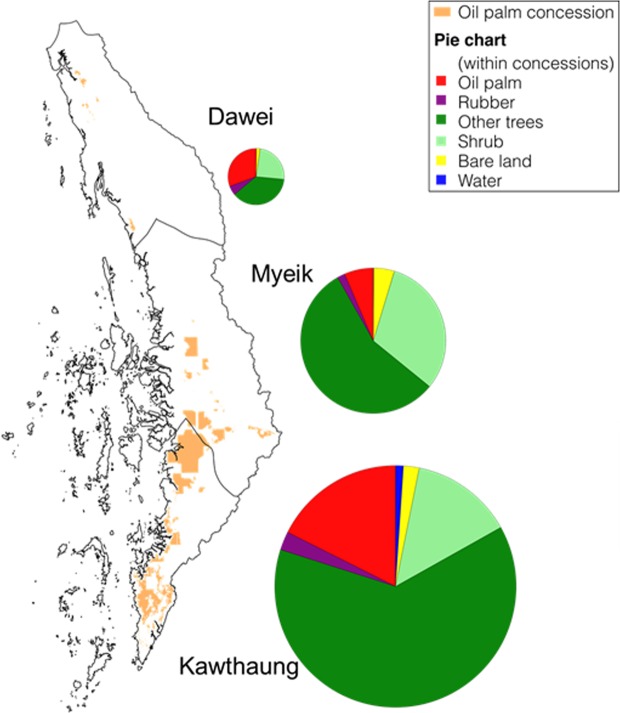
Planted area estimates by district. The pie chart shows land use of concession areas in each district, with the size corresponding to the size of total concessions.

In 2018, oil palm plantations (mature oil palm trees >4 years) covered approximately 75 kha (69–81 kha range at 95% confidence) of the Tanintharyi region (Table 2). Less than 70% (45–52 kha) of the oil palm plantations are within the concession areas, with approximately 25 kha planted outside (using the most recently available palm oil concession boundaries)^42^. By district, the southernmost district Kawthaung has the largest oil palm concession areas, with 63% of oil palm in the region planted in Kawthaung (Fig. 2). The pattern of planting differed between the regions, with 84% of oil palm in Kawthaung planted within concession areas, compared to 34–35% in northern Dawei and Myeik districts. In total, only 6% of concession areas in the Myeik district were planted with oil palm, with the remainder made up of rubber (2%) and other trees (56%) (Fig. 2). Dawei and Kawthaung districts had higher stocking rates, with 31% and 18% of concessions planted with oil palm, and 5% and 3% with rubber, respectively (Fig. 2).

**Table 2:** Area estimates by class (ha).

(ha)	Oil palm	Rubber	Other trees	Shrub	Bare	Water
Total	75,160	111,122	3,056,373	667,310	173,866	46,080
95% confidence	69,212–81,108	106,312–115,689	3,047,756–3,065,084	66,0851–67,3505	171,308–175,958	44,107–47,556
Within concession	49,276	7,771	195,246	60,853	9,013	2,547
95% confidence	45,119–52,932	7,392–8,007	194,523–195,833	59,897–61,494	8,723–9,142	2,456–2,630

Other trees include forests, tree plantations and tree-crops other than oil palm and rubber such as areca (betel nut) or cashew nut trees. Shrub includes grassland, open canopy, and young and low vegetation. Bare land includes sand. See Fig. S2 for examples.

**Figure 3:**
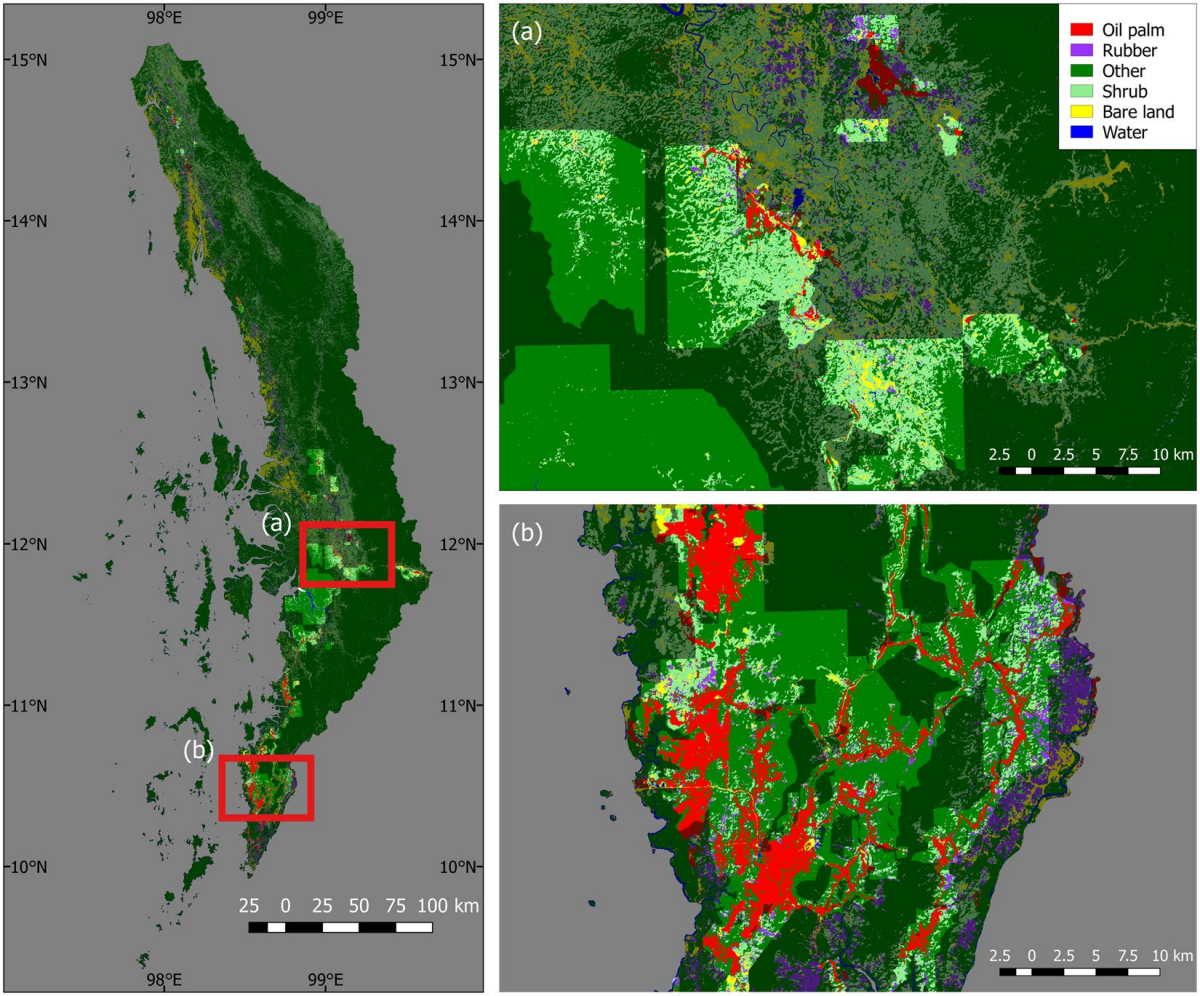
Classification results: map of the region by class (concession areas brighter).

The area shown in Fig. 3(a) in the Myeik district contains large areas of unplanted concessions, compared to the area in Fig. 3(b) in the Kawthaung district, where the oil palm plantations are concentrated. While some oil palm plantations located outside of concessions are an extension of or in close proximity to nearby concessions, others do not seem to have any relationship with concession boundaries. It also appears that oil palm plantations tend to be found along the roads (Fig. 3(b)).

**Figure 4:**
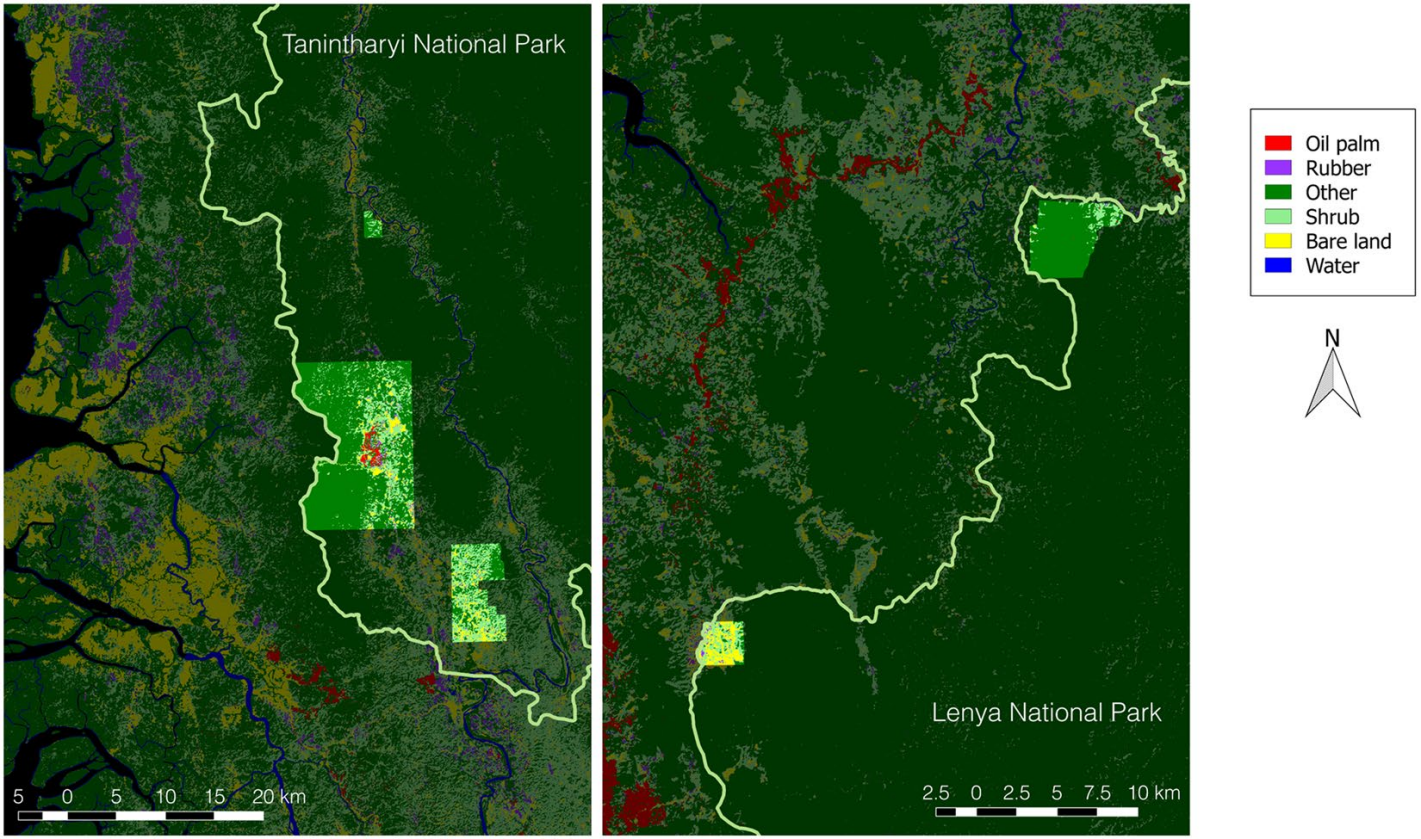
Classification results: concessions inside proposed national parks (concession areas brighter).

Some concessions overlap with proposed national parks (Tanintharyi National Park and Lenya National Park, both proposed in 2002). Within national park boundaries, approximately 4% of concessions (about 1,000 ha) are planted with oil palm or rubber plantations (2% each) (Fig. 4), a considerably smaller proportion than 15% of concessions planted with oil palm in the region. Bare land covers about 6% of concession areas in the national parks, compared to 1% in the entire national parks. This leaves 65% (or 90% including shrub) of the concessions in the national parks which have not been planted with oil palm or rubber.

**Figure 5:**
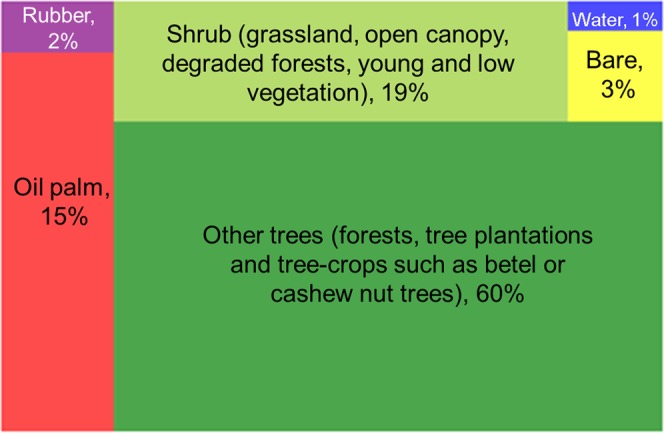
Land use of oil palm concession area. Other trees include forests, tree plantations and tree-crops other than oil palm and rubber such as areca (betel nut) or cashew nut trees. Shrub includes grassland, open canopy, and young and low vegetation. Bare land includes sand.

The rubber plantations (mature rubber trees >6 years) account for approximately 111 kha in the region, which is 1.5 times larger than the total areas of oil palm plantations (Table 2). The rubber is mostly located outside the oil palm concessions: only about 7 kha of this is found within the concessions, making up just 2% of oil palm concession areas (Table 2 and Fig. 5). Most of these cases (72%) are in the southern tip of the Kawthaung district, where a large portion of the land is used for crops, and oil palm and rubber are often planted next to each other.

In total, only 15% of concession areas are planted with oil palm while 60% (approximately 195 kha) are classified as “other trees” (this includes all other vegetation types, i.e. forests and tree-crops other than oil palm and rubber, such as betel or cashew nut trees (Fig. 5).

## Discussion

The results show that current oil palm plantations are much smaller than what has been reported to the government: 35% of concession areas (140 kha out of 401 kha) were reported to have been planted in 2015^18^, while in our study 14 to 16% of the oil palm concessions (45–52 kha out of 324 kha) remain planted in the late 2018 and the beginning of 2019. Our estimates for oil palm plantations in the region are much smaller than in other studies (75 kha as compared to 136 kha^25^ for 2016 and 125 kha^47^ for 2018. However, their methodologies differ from ours, most notably on the inclusion of red edge bands as well as the amount of reference data. We argue that other studies experienced difficulty in distinguishing oil palm from other tree crops or vegetation, which also resulted in lower accuracy rates.

A number of reasons could explain the discrepancy from the government data. Firstly, it could be partially explained by the 25 kha of oil palm that are planted outside of concession areas. If included, the total would become 23% of the current concession areas (69–81 kha). Secondly, the four-year difference between the two datasets may affect this difference: some old oil palm may have been cut down and plantations abandoned or replaced with other crops. This is possible given reports of declining oil palm business, especially in the north of the region, where the climatic conditions are less favourable^18,28^. This decline may be compounded by the limited number of processing facilities which are owned by a few companies, the availability of cheap oil palm imports, lower yields and poor quality of the palm oil^18,48^. Finally, it is possible that there may be over-reporting of planted areas in the past, as the progress was previously monitored by the government^49^, and there may have been political reasons for the companies to over-report these figures.

While large areas of oil palm appear to have been planted outside of concession areas, the boundaries may have been unclear to companies, and some concessions have been cancelled or updated based on planted areas. Nevertheless, it is extremely important to clarify and demarcate the concession boundaries and start enforcing them in practice. Furthermore, there are oil palm concessions inside proposed national parks. The Tanintharyi National Park in particular has about 1,000 ha planted with oil palm and rubber. It is crucial to examine the suitability of remaining concession areas as oil palm plantations.

The rubber plantations are estimated to cover a larger area than the oil palm plantations. Our estimates (111 kha) are relatively consistent with the reported data (138 kha) in 2015–2016 and the study by Connette *et al*. (127 kha). The defoliation phase of rubber trees may have contributed to the difference, as our optical satellite data was collected during the dry season (November to January, when there are fewer clouds so optical satellite data is more likely to be available), which means some rubber could have been missed^32–34^. The prevalence of rubber means that even if demand for oil palm slows, depending on the location, deforestation could still occur due to demand for rubber or other crops, as evidenced in the rubber planted in oil palm concessions.

Based on our study, rubber is the dominant crop in the region, while oil palm, although still the largest crop within the concessions, is planted much less than expected, leaving an extensive area available for other uses such as conservation or communities’ livelihood needs.

The unconverted portions of the concessions represent a significant risk clearly, as they could legally be cleared at any time, but also an important opportunity for conservation and the global climate. These 195,246 hectares have an average aboveground carbon stock of 209.3 Mg C per ha^−1^ ^50^, which means that clearing them would release at least 149.9 Tg CO_2_e to the atmosphere, and likely more as this estimate ignores belowground and soil carbon pools. To put this in perspective, this is almost as much as the annual carbon emissions of the Netherlands in 2017, and over five times more than Myanmar’s 2017 emissions from burning fossil fuels and cement manufacture^51^. Although these forests are included in Myanmar’s Forest Reference Level under REDD+, the risks are calculated based on historical changes in forest cover at the national level. Clearly making space legally for the rescinding of such concessions could greatly reduce future emissions from the region and promote the protection of intact forests^52,53^. This is especially relevant given the changing climate in this region, which could make Myanmar increasingly viable as a place to grow palm oil, just as Indonesia decreases in viability^24^.

## Supplementary information

Supplementary information

## Data Availability

All data and code that support the figures and tables are available; on publication these will be uploaded to an open data repository (See Supplementary Material).
