# Supplementary material for: Oil palm concessions in southern Myanmar consist mostly of unconverted forest
Source: Sci Rep. 2019 Aug 15;9:11931. doi: 10.1038/s41598-019-48443-3 (PMC6695397; doi:10.1038/s41598-019-48443-3)
Supplement: Supplementary file 1 — Supplementary information [file 41598_2019_48443_MOESM1_ESM.pdf]

Supplementary information for

Oil palm concessions in southern Myanmar consist mostly of unconverted forest

Keiko Nomura[1*], Edward T.A. Mitchard[1], Genevieve Patenaude[1], Joan Bastide[2], Patrick Oswald[3], and Thazin Nwe[4]

[1] University of Edinburgh, School of GeoSciences, Edinburgh, EH9 3FF, United Kingdom

[2] Centre for Development and Environment (CDE), University of Bern, Hallerstrasse 10, 3012 Bern, Switzerland

[3] OneMap Myanmar, No. E2 New University Yeik Mon, New University Avenue, Bahan Township, Yangon, Myanmar

[4] Biodiversity Conservation Group, Center for Integrative Conservation, Xishuangbanna Tropical Botanical Garden, Chinese Academy of Sciences, Menglun, Xishuangbanna, Yunnan, 666303, China

*keiko.nomura@ed.ac.uk

Data, code, map

See https://github.com/nkeikon/tanintharyi for the codes and reference data used for this study.

Classification, reference data, and accuracy

The classification was conducted by separating the southernmost township, Kawthaung in the Kawthaung district (Figure S1), because its different characteristics meant that significant errors resulted when the two were considered together. The two maps were then mosaicked together for display purposes and the figures in the main paper, but accuracy and areas are considered separately below.

The areas were estimated by correcting bias, thus adding up the number of pixels for each class in the output map will not produce the same areas as those shown in the tables and in the text. See Table S3 and S4.

Table S1. Reference data (20x20m pixel count)

	Area 1	Area 2	Total
Oil palm	21,564	11,381	32,945
Rubber	6,863	6,521	13,384
Other trees	56,520	37,866	94,386
Shrub	18,399	2,186	20,585
Bare land	1,301	1,083	2,384
Water	6,985	247	7,232
Total	111,632	59,284	170,916

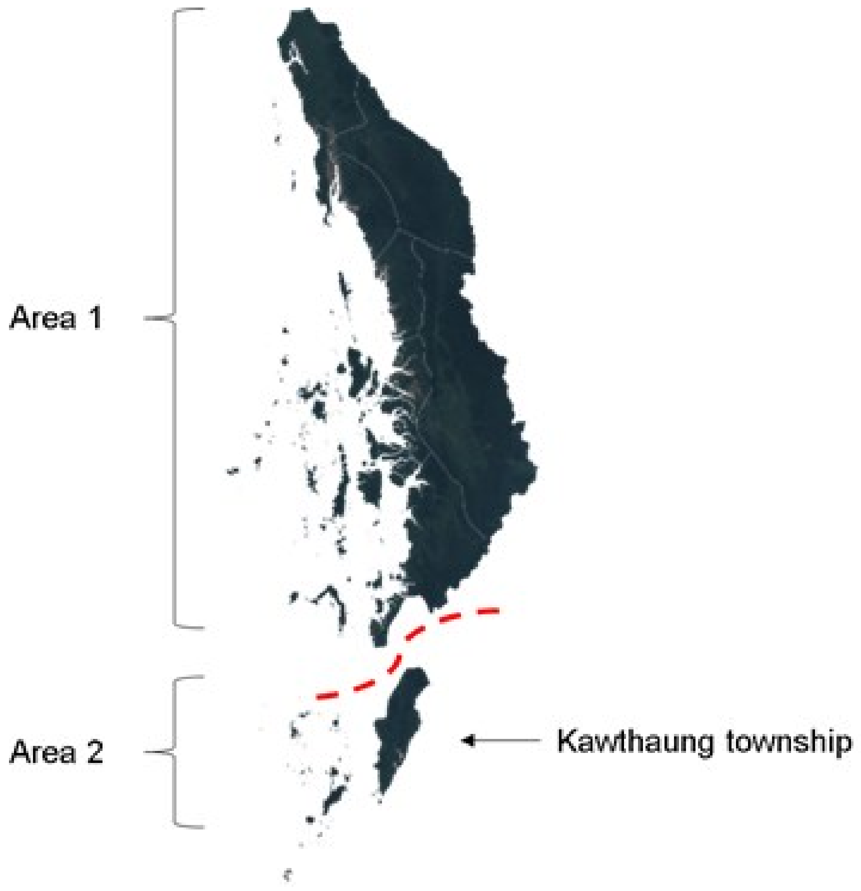

Figure S1. Two areas for classification

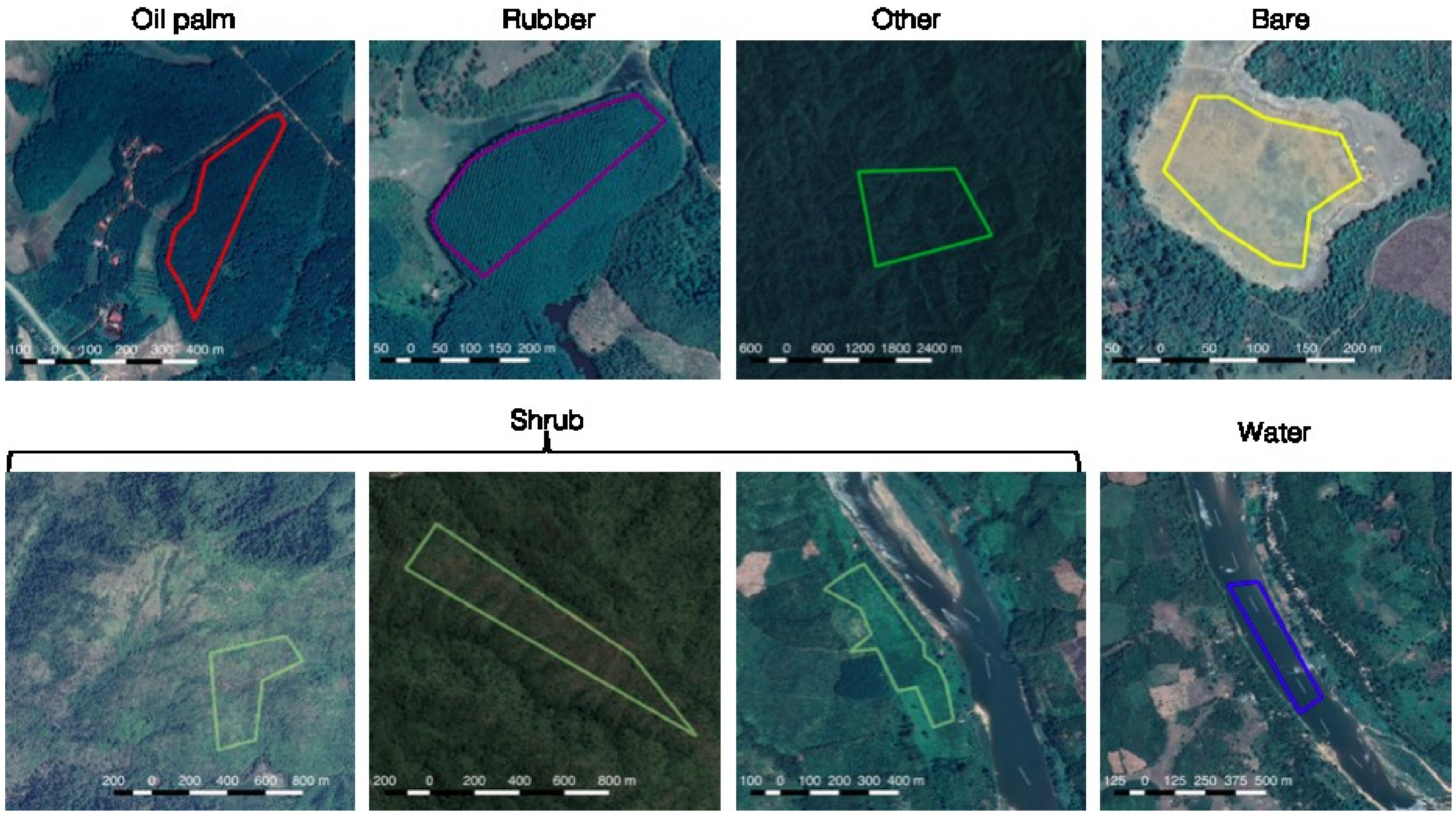

Figure S2. Examples of reference data for each class. Images were delineated as polygons using QGIS 2.18.20. (Sources: Google Earth Pro 7.3.2.5776. Tanintharyi, Myanmar, DigitalGlobe 2019.)

Table S2. Error matrices

Area 1

Class	Oil palm	Rubber	Other	Shrub	Bare land	Water	User's accuracy	Producer's accuracy	Overall accuracy
**Oil palm**	9,815	28	956	44	1	0	91%	96%	94%
**Rubber**	6	2,790	246	413	0	0	81%	95%	
**Other**	445	29	27,394	520	0	0	96%	94%	
**Shrub**	2	91	567	8,500	11	0	93%	89%	
**Bare land**	0	0	0	22	649	1	97%	98%	
**Water**	0	0	0	3	2	3,487	100%	100%	
**Total**	10,268	2,938	29,163	9,502	663	3,488			

Area 2

Class	Oil palm	Rubber	Other	Shrub	Bare land	Water	User's accuracy	Producer's accuracy	Overall accuracy
**Oil palm**	4,882	27	862	16	4	0	84%	96%	94%
**Rubber**	0	3,017	187	29	4	0	93%	91%	
**Other**	224	70	18,520	29	2	0	98%	94%	
**Shrub**	1	194	54	868	1	0	78%	92%	
**Bare land**	0	9	8	5	531	0	96%	98%	
**Water**	0	0	8	0	0	117	94%	100%	
**Total**	5,107	3,317	19,639	947	542	117			

Table S3. Bias-corrected area estimation and error matrix (Area 1) [1,2]

Error matrix, sample counts

		Reference						Total	Pixels	W_i
		Oil palm	Rubber	Other	Shrub	Bare	Water			
Map	Oil palm	21,044	1	12	2	-	-	21,058	711,381	0.007
	Rubber	7	6,401	11	24	1	-	6,444	1,881,140	0.019
	Other	500	210	56,177	369	-	9	57,265	75,163,984	0.753
	Shrub	37	236	241	17,962	34	9	18,520	16,705,549	0.167
	Bare	2	1	1	1	1,257	3	1,265	4,322,056	0.043
	Water	0	-	0	0	-	6,969	6,969	1,028,425	0.010
Total		21,590	6,849	56,442	18,358	1,292	6,990	111,520	99,812,534	1.000

Error matrix, estimates area proportions

		Reference						Total	Pixels	W_i
		Oil palm	Rubber	Other	Shrub	Bare	Water			
Map	Oil palm	0.0071	0.0000	0.0000	0.0000	0.0000	0.0000	0.0071	711,381	0.007
	Rubber	0.0000	0.0187	0.0000	0.0001	0.0000	0.0000	0.0188	1,881,140	0.019
	Other	0.0066	0.0028	0.7387	0.0049	0.0000	0.0001	0.7531	75,163,984	0.753
	Shrub	0.0003	0.0021	0.0022	0.1623	0.0003	0.0001	0.1674	16,705,549	0.167
	Bare	0.0001	0.0000	0.0000	0.0000	0.0430	0.0001	0.0433	4,322,056	0.043
	Water	0.0000	0.0000	0.0000	0.0000	0.0000	0.0103	0.0103	1,028,425	0.010
	Total	0.0141	0.0236	0.7410	0.1673	0.0433	0.0106	1.0000	99,812,534	1.000
	Area [pix]	1,409,392	2,358,747	73,961,429	16,698,297	4,326,248	1,058,420	99,812,534		
	Area [ha]	54,794	91,211	2,868,109	646,200	167,452	41,099	3,992,501		
	S(Area)	0.0003	0.0002	0.0005	0.0003	0.0001	0.0001			
	S(Area) [ha]	2,710	2,129	4,071	2,970	980	681			
	95% CI [ha]	5,311	4,173	7,980	5,822	1,920	1,335			
	User's	100%	100%	98%	97%	100%	100%			
	Producer's	49%	80%	100%	97%	100%	98%			
	Overall	98%								

Table S4. Bias-corrected area estimation and error matrix (Area 2)[1,2]

Error matrix, sample counts

		Reference						Total	Pixels	W_i
		Oil palm	Rubber	Other	Shrub	Bare	Water			
Map	Oil palm	10,551	0	35	0	0	0	10,586	421,187	0.063
	Rubber	20	6,296	26	101	8	0	6,451	492,918	0.074
	Other	750	186	37,768	76	5	39	38,824	4,945,663	0.741
	Shrub	11	16	4	2,010	8	0	2,050	532,723	0.080
	Bare	7	2	1	2	1,059	0	1,071	162,405	0.024
	Water	0	0	0	0	0	209	209	122,339	0.018
	Total	11,340	6,500	37,833	2,189	1,080	248	59,191	6,677,236	1.000

Error matrix, estimates area proportions

		Reference						Total	Pixels	W_i
		Oil palm	Rubber	Other	Shrub	Bare	Water			
Map	Oil palm	0.0629	0.0000	0.0002	0.0000	0.0000	0.0000	0.0631	421,187	0.063
	Rubber	0.0002	0.0720	0.0003	0.0012	0.0001	0.0000	0.0738	492,918	0.074
	Other	0.0143	0.0036	0.7205	0.0015	0.0001	0.0007	0.7407	4,945,663	0.741
	Shrub	0.0004	0.0006	0.0002	0.0782	0.0003	0.0000	0.0798	532,723	0.080
	Bare	0.0002	0.0000	0.0000	0.0000	0.0241	0.0000	0.0243	162,405	0.024
	Water	0.0000	0.0000	0.0000	0.0000	0.0000	0.0183	0.0183	122,339	0.018
	Total	0.0780	0.0763	0.7212	0.0809	0.0246	0.0191	1.0000	6,677,236	1.000
	Area [pix]	520,961	509,179	4,815,694	540,096	163,994	127,312	6,677,236		
	Area [ha]	20,366	19,911	188,265	21,110	6,413	4,981	267,089		
	S(Area)	0.0005	0.0003	0.0006	0.0003	0.0001	0.0001			
	S(Area) [ha]	325	201	373	191	88	72			
	95% CI [ha]	637	394	731	374	172	140			
	User's	100%	98%	97%	98%	99%	100%			
	Producer's	81%	94%	100%	97%	98%	96%			
	Overall	98%								

References

1. Olofsson, P., Foody, G. M., Stehman, S. V. & Woodcock, C. E. Making better use of accuracy data in land change studies: Estimating accuracy and area and quantifying uncertainty using stratified estimation. *Remote Sensing of Environment* **129**, 122–131 (2013).
2. *Open source tutorials on remote sensing data analysis : beeoda/tutorials*. (Boston Education in Earth Observation Data Analysis, 2019).
